# Altered upper respiratory tract microbiota in laryngeal cough attributed to lung yin deficiency and the modulatory effects of Yangyin Qingfei Oral Liquid

**DOI:** 10.3389/fmicb.2025.1592294

**Published:** 2025-06-26

**Authors:** Fei Yu, Jing Jin, Xinlai Jin, Xuchun Ding, Yafang Lou

**Affiliations:** ^1^Department of Respiratory and Critical Care Medicine, Hangzhou TCM Hospital Affiliated to Zhejiang Chinese Medical University, Hangzhou, China; ^2^Graduate School, Zhejiang Chinese Medical University, Hangzhou, China

**Keywords:** upper respiratory tract microbiota, laryngeal cough, Yangyin Qingfei Oral Liquid, 16S rDNA metagenomic sequencing, lung yin deficiency

## Abstract

**Objective:**

This study investigates changes in upper respiratory tract microbiota in laryngeal cough patients with lung yin deficiency using high-throughput sequencing of the 16S rDNA gene. It also examines the modulatory effects of Yangyin Qingfei Oral Liquid (YYQFOL).

**Methods:**

We included 100 laryngeal cough patients and 65 healthy subjects, collecting throat swab samples for microbiota comparison. Patients were randomly assigned to a control group receiving methoxyphenamine capsules and an experimental group receiving YYQFOL and methoxyphenamine for 10 days. We assessed changes in microbiota, symptom scoring, and Leicester Cough Questionnaire (LCQ) results. Each group was divided into responders (R) and non-responders (NR).

**Results:**

Patients with laryngeal cough had significantly lower microbial abundance and diversity than healthy subjects (*p* < 0.05). After treatment, symptom scores and LCQ results improved significantly (*p* < 0.05), with responders in the experimental group (ER) showing significantly better improvement than those in the responders in the control group (CR) (*p* < 0.05). Post-treatment, the experimental group saw a significant reduction in *Streptococcus*, *Haemophilus*, and other genera, while *Veillonella* increased (*p* < 0.05).

**Conclusion:**

Laryngeal cough patients with lung yin deficiency are imbalanced in the upper respiratory tract microbiota. Treatment with methoxyphenamine and YYQFOL improves microbiota composition and alleviates symptoms.

## 1 Introduction

Laryngeal Cough refers to chronic cough triggered by structural or functional abnormalities of the larynx, primarily caused by chronic inflammation in the laryngopharyngeal region (Sundar et al., 2024). This inflammation induces hypersensitivity of the laryngeal mucosa or nerves, leading to a lowered cough reflex threshold (Sundar et al., 2024). Consequently, even minor stimuli can provoke persistent dry coughing. Unlike post-infectious cough from common respiratory infections, laryngeal cough typically lacks evidence of pulmonary pathology. Diagnosis requires exclusion of other causes through laryngoscopy and detailed clinical history (Asthma Group of Chinese Thoracic Society, 2022). In Traditional Chinese Medicine (TCM) theory, lung yin deficiency denotes a pathological state of lung yin depletion causing deficiency-heat, presenting with paroxysmal unproductive cough, itchy or sore throat, and dry throat. The tongue appears red with scant coating, and the pulse is thready and rapid (National Administration of Traditional Chinese Medicine, 1994). A total of 539 outpatients from the First Affiliated Hospital of Guangxi University of Chinese Medicine were surveyed via questionnaire during January 2013–October 2016. Notably, the pattern of Yin Deficiency with Exuberant Fire accounted for 3.2% of the total cases (Zhang et al., 2017).

Recent studies have demonstrated that changes in the respiratory tract microbiota are closely linked to respiratory diseases (Hilty et al., 2010; Wypych et al., 2019; Chai et al., 2022). Research has indicated that the upper respiratory tract microbiota undergoes notable changes in patients with laryngeal cough (Dąbrowska et al., 2020). According to a systematic review of 10 eligible studies, the healthy larynx harbors a diverse microbial community dominated by *Streptococcus*, *Cloacibacterium*, *Prevotella*, and *Helicobacter*. In benign laryngeal diseases, microbial diversity decreases, with *Streptococcus* dominating the community. Laryngeal squamous cell carcinoma is associated with increased microbial diversity and an abundance of *Fusobacterium*, highlighting the role of dysbiosis in malignant conditions (Sragi et al., 2024).

Yangyin Qingfei Oral Liquid (YYQFOL) is a traditional Chinese patent medicine derived from the Yangyin Qingfei Decoction, originally described in *Chonglou Yuyao*, a work by the famous Qing Dynasty physician Zheng Meijian (Zhang et al., 2024). The formula contains ingredients such as Rehmannia Glutinosa, *Ophiopogon Japonicus*, Scrophularia Ningpoensis Hemsl, Fritillariae Thunbergii Bulbus, Radix Paeoniae Alba, Cortex Moutan, Corn Mint, and Glycyrrhiza Uralensis Fisch. Yangyin Qingfei Decoction is well-known for its ability to nourish and clear the lungs and is particularly recognized for its effectiveness in treating diphtheria (Tang et al., 2022). YYQFOL, which is based on this original prescription, contains the same eight ingredients. Clinically, it is used to treat chronic pharyngitis, pharyngeal neurosis, and chronic bronchitis, which are characterized by a dry and sore throat and a cough with little or no sputum. After years of clinical validation, this prescription has proven to be effective with minimal side effects. Previous clinical studies indicate that YYQFOL has beneficial effects on laryngeal cough, alleviating symptoms and improving patients’ quality of life (Pan et al., 2018).

Laryngeal cough can significantly impair patients’ quality of life. TCM treatments for laryngeal cough have been shown to relieve clinical symptoms, improve quality of life, and demonstrate positive clinical effects, highlighting their advantages and unique characteristics (Minamizawa et al., 2006). In this study, we examined the variations in the upper respiratory microbiota of patients with laryngeal cough of lung yin deficiency and explored the modulatory effects of YYQFOL on the upper respiratory tract microbiota in individuals suffering from laryngeal cough.

## 2 Materials and methods

### 2.1 Inclusion criteria

The inclusion criteria were as follows: (1) According to the chronic pharyngitis classification standard launched by *Otorhinolaryngology Head and Neck Surgery* published by the People’s Medical Publishing House in 2018; (2) Diagnosed as lung yin deficiency; (3) Aged 18–65, both genders are welcome; (4) Without abnormalities in vital signs, physical examination, complete blood count (CBC), biochemical blood test, and chest X-ray; (5) All patients with good compliance signed the informed consent form.

### 2.2 Exclusion criteria

The exclusion criteria were as follows: (1) Patients with bronchitis, bronchial asthma, bronchial foreign body, pneumonia, lung tumor, upper airway cough syndrome (UACS/PNDS) and Gastroesophageal Reflux Disease (GERD); (2) Patients subjected to serious diseases such as heart, brain and hematopoietic system; (3) Pregnant or lactating women, and mental patients; (4) Allergic individuals; (5) Patients with recent antibiotic use within a week; (6) Poor dependency.

### 2.3 Subjects

Between April and October 2024, 100 patients diagnosed with laryngeal cough attributed to lung yin deficiency were enrolled at Hangzhou Hospital of Traditional Chinese Medicine, Zhejiang Chinese Medical University. All patients met the aforementioned diagnostic criteria. All patients met the established diagnostic criteria and exhibited measurable disease severity, assessed using a symptom assessment scale and the Leicester Cough Questionnaire (LCQ). The participants were randomly divided into two groups for observation.

During the observation period, two patients dropped out from the experimental and two from the control groups, leaving 96 patients in the final analysis. In addition, 65 healthy adults were selected as control subjects. These healthy controls had no respiratory diseases and had not taken antibiotics within the week before the study. The study received approval from the Ethics Committee of Hangzhou Hospital of TCM. All patients were informed about the study and provided their consent to participate. All procedures adhered to the *Declaration of Helsinki*.

### 2.4 Treatment

The laryngeal cough group (LC) received compound methoxyphenamine capsules produced by Daiichi Sankyo Pharmaceutical (Shanghai) Co., Ltd., upon enrollment. The patients in the experimental group were also given YYQFOL capsules produced by Songlu Pharmaceutical Co., Ltd. (Zhejiang, China). Patients in the experimental group took 10 mL of the YYQFOL medication three times daily, while patients in both the treatment and control groups took two capsules of the methoxyphenamine three times a day for a 10-day treatment course. The health control group (HC), which consisted of healthy controls, did not receive any medication or treatment during the study period; this group served as a baseline for comparison with the other groups. Disease evaluations and sample collections were performed before and after the treatment. The pharyngeal swab samples from the HC group were collected only at the time of enrollment.

### 2.5 Specimen collection

All research participants were instructed to open their mouths with their heads tilted backward. A sampler used a sterile flocked swab to gently obtain samples from the posterior and lateral walls of the pharynx three times, avoiding contact with the teeth, oral mucosa, and tongue. Once collected, the samples were stored in a refrigerator at −80°C until they were submitted for testing.

**Figure 1:**
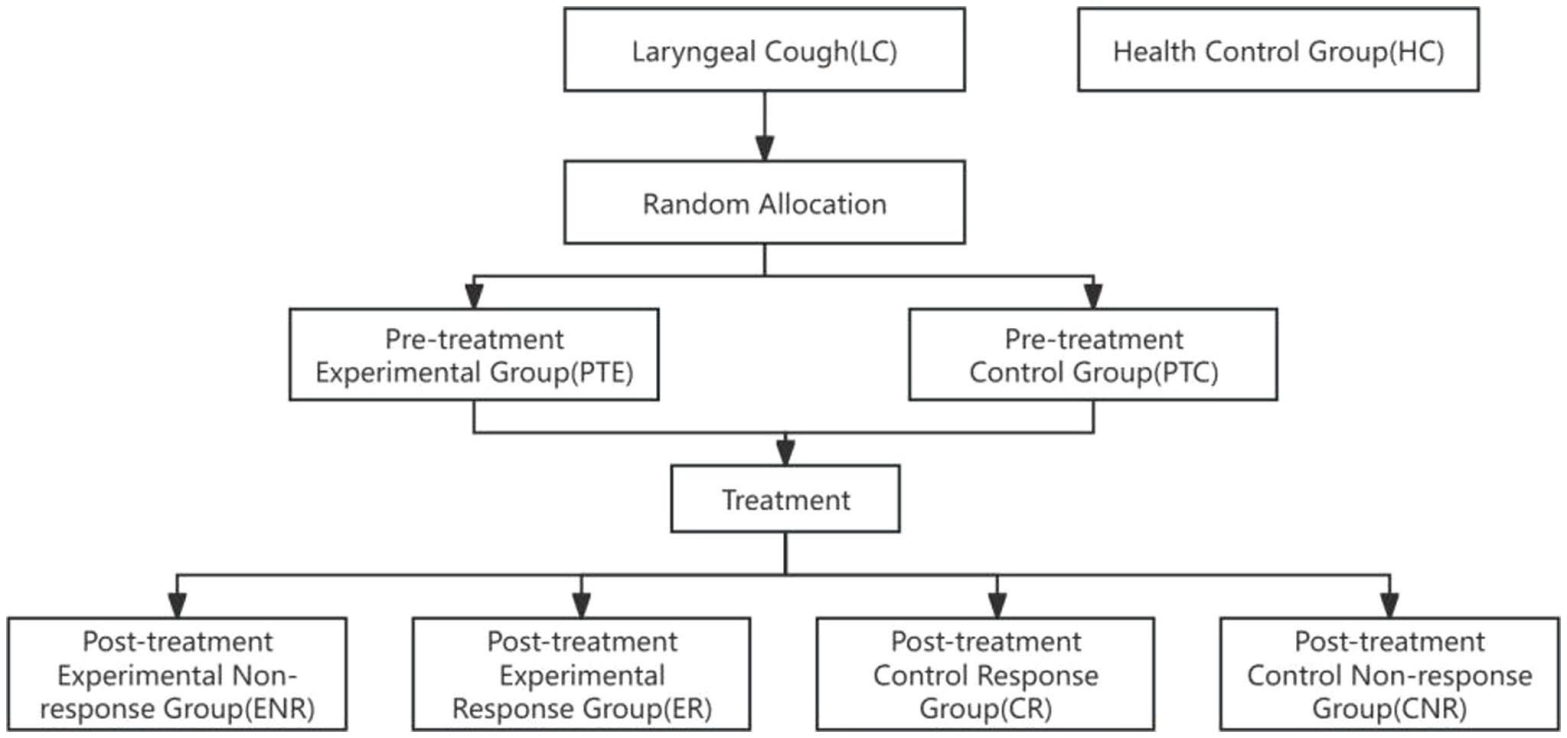
Flow chart. Participants were initially categorized into two groups: Laryngeal Cough (LC) and Health Control Group (HC). Random allocation was performed to divide the LC group into the Pre-treatment Experimental Group (PTE) and the Pre-treatment Control Group (PTC). Following treatment, the experimental group was further subdivided into the Experimental Non-response Group (ENR) and the Experimental Response Group (ER), while the control group was divided into the Control Response Group (CR) and the Control Non-response Group (CNR). The diagram illustrates the progression of participants through the study phases.

A total of 261 valid throat swab samples were collected and categorized as follows: Healthy control group (HC): *n* = 65, Pre-treatment control group (PTC): *n* = 50, Post-treatment control response group (CR): *n* = 40, Post-treatment control non-response group (CNR): *n* = 8, Pre-treatment experimental group (PTE): *n* = 50, Post-treatment experimental response group (ER): *n* = 44, Post-treatment experimental non-response group (ENR): *n* = 4, as shown in Figure 1.

### 2.6 Indicators for assessing therapeutic efficacy

#### 2.6.1 Symptom and sign scoring table

Based on the diagnostic criteria for lung heat and yin deficiency related to chronic pharyngitis, as outlined in the *Diagnostic and Therapeutic Criteria for TCM*, along with the clinical diagnostic criteria detailed in the third edition of *Otolaryngology Head and Neck Surgery*, we developed a symptom assessment scale. This scale includes 10 indicators: cough, sparse sputum, dry throat, itchy throat, sore throat, sensation of a foreign body in the pharynx, dry mouth, dry stool, lymphoid follicular hyperplasia of the posterior pharyngeal wall, and pharyngeal mucosal congestion. Each indicator is scored as zero, two, four, or six points, corresponding to the absence, mild, moderate, or severe severity levels. Assessments were conducted before and after treatment.

#### 2.6.2 Leicester cough questionnaire

The LCQ is a primary assessment tool used to evaluate the severity of cough and its impact on a person’s life. The questionnaire consists of 19 items covering three key domains: physiological, social, and psychological aspects of health. It is designed so that higher scores indicate better health status. In this study, LCQ scores were assessed before and after treatment to evaluate the effectiveness of the intervention and track changes in the patient’s condition over time. By comparing pre-treatment and post-treatment scores, the study aimed to assess any improvements or potential worsening in the patient’s overall health and well-being related to coughing.

#### 2.6.3 Criteria for evaluating curative effect

The criteria to evaluate the curative effect were based on the *Guiding Principles for Clinical Research of New TCM* and the Standards for *Diagnosis and Therapeutic Effect of TCM Syndromes* regarding chronic pharyngitis. Patients were considered cured if the symptom assessment score decreased by ≥95%, and major symptoms such as itchy throat, cough, dry throat, lymphoid follicular hyperplasia of the posterior pharyngeal wall, and pharyngeal mucosal congestion either disappeared or nearly disappeared, with normal recovery of throat signs. A significant improvement was noted if the score decreased by ≥70%, indicating substantial improvement in symptoms and throat signs. An effective result was seen if the score decreased by ≥30%, reflecting mild relief in symptoms and improvement in throat signs. If the score decreased by <30%, with less than 30% symptom relief and no significant changes in symptoms and signs, the treatment was considered ineffective. According to these criteria, patients in this study were classified as responders (R) if their score improved by ≥30% or non-responders (NR) if the score improved by <30%.

### 2.7 Upper respiratory tract microflora detection

#### 2.7.1 16S rDNA sequencing

The nucleic acid extraction of pharyngeal swab specimens was performed according to the protocol of the DNA Extraction Kit (Bacterial Genome Extraction Kit, TIANGEN DP302-02). Primers were designed based on the V3-V4 region of the 16S rRNA gene, with sequencing adapters added to the primer ends for PCR amplification. The amplification products were purified using AMPure XT beads (Beckman Coulter Genomics, Danvers, Massachusetts, United States) and quantified by Qubit (Invitrogen, United States). Purity evaluation was conducted using the Agilent 2100 Bioanalyzer (Agilent, United States) and the Illumina library quantification kit (Kapa Biosciences, Woburn, Massachusetts, United States). Qualified sequencing libraries for sequencing were gradient-diluted, pooled, and denatured, followed by paired-end sequencing with a read length of 2 × 250 bp on the NovaSeq 6000 sequencer (PE250 mode) to construct the sequencing library, provided by LC-Bio Technology Co., Ltd., Hangzhou, China.

#### 2.7.2 Bioinformatics analysis

**Figure 2:**
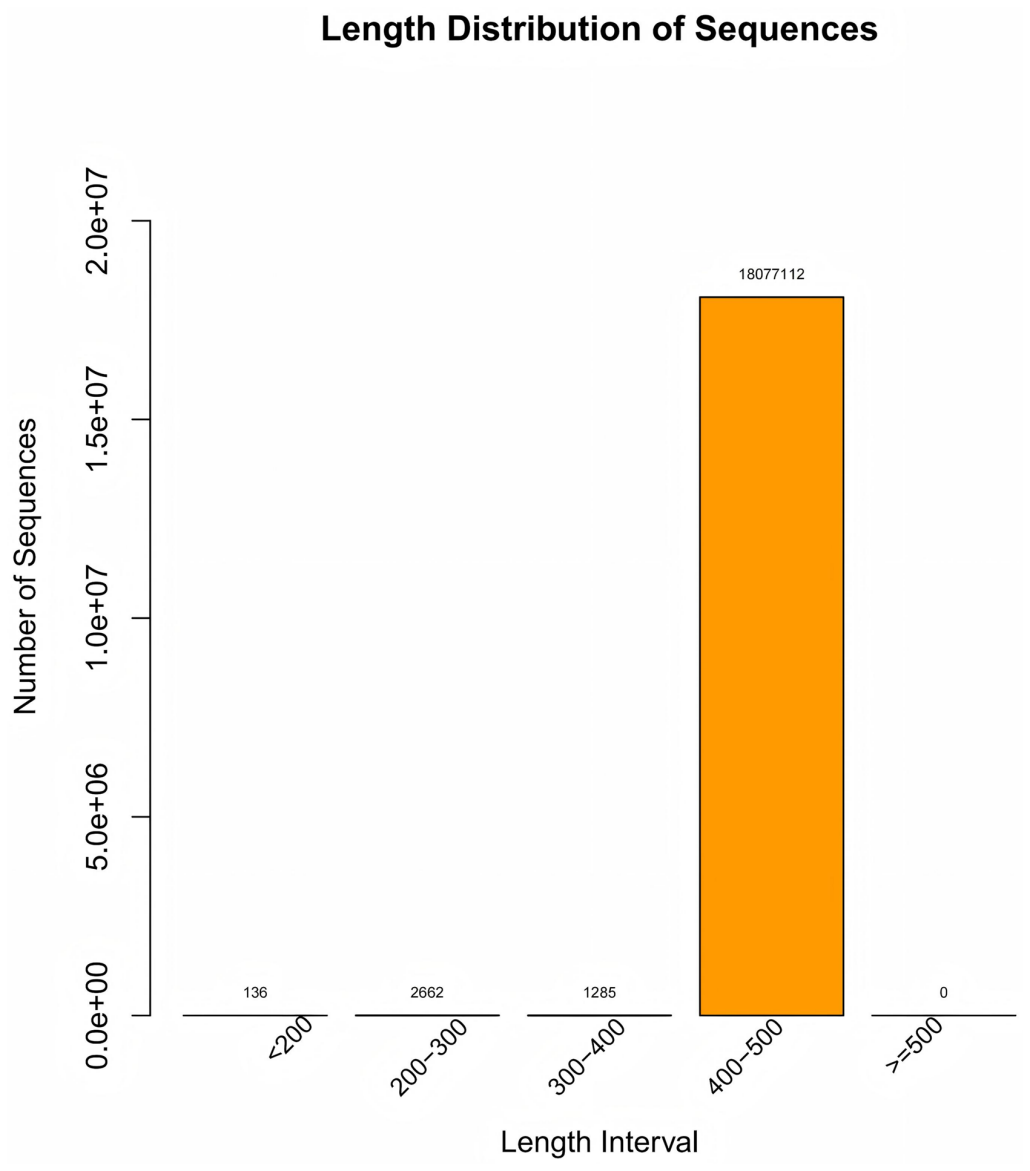
Length distribution of sequence. The “Length Distribution of Sequence” diagram illustrates the quantity of sequences across various length ranges, with the 400–500 bp interval containing 18,077,112 sequences—a crucial metric for sample quality control.

**Figure 3:**
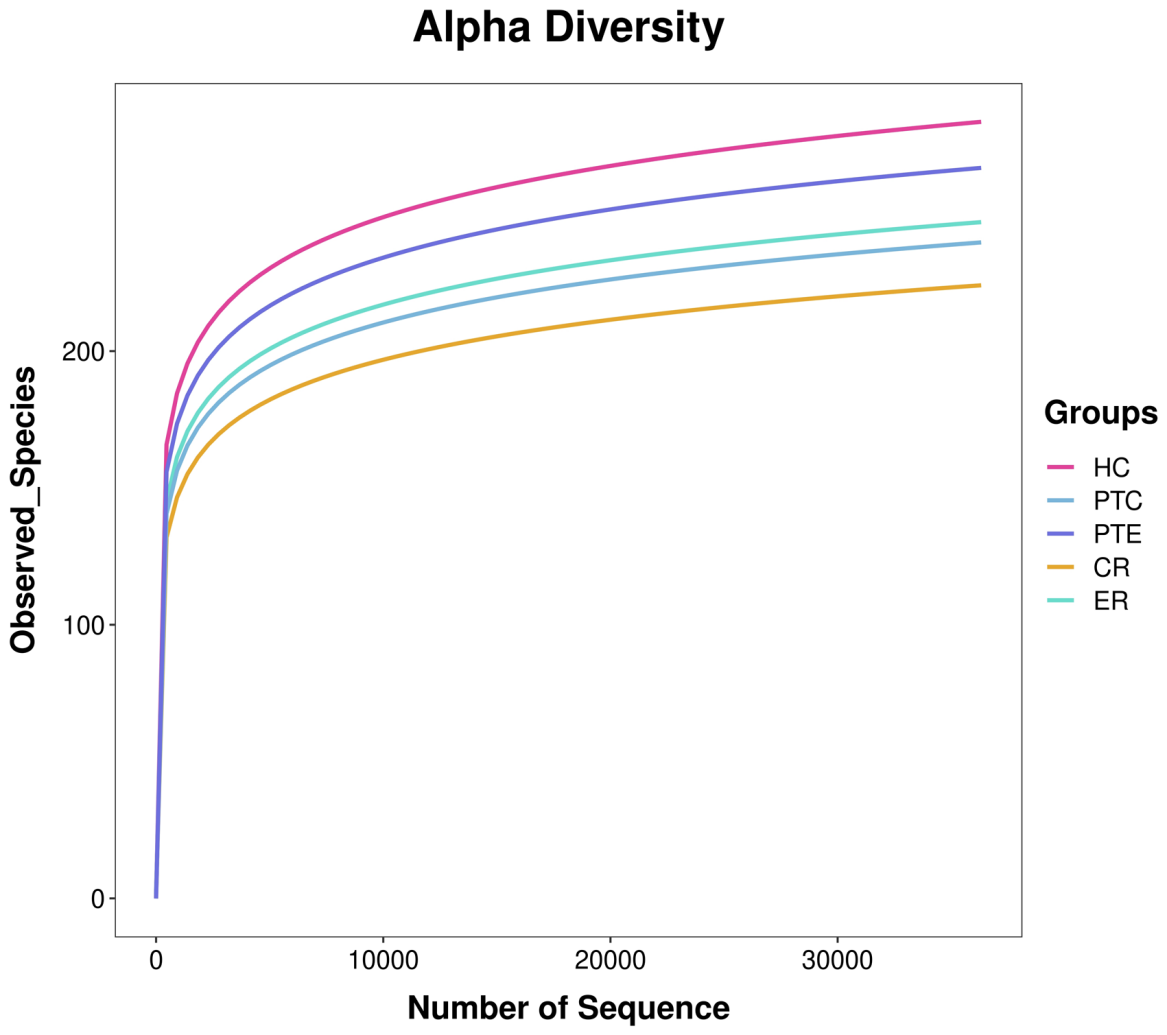
Species accumulation curve. The x-axis represents the number of randomly sampled sequences, while the y-axis indicates the corresponding ASV count. The slope variation of the rarefaction curve reflects the saturation status of sequencing depth.

The original data Raw Data obtained after sequencing was spliced and filtered to yield high-quality Clean Data. Through quality control steps such as removing adapters, primers, and low-quality sequences using QIIME2 D2DA2 (Divisive Amplicon Denoising Algorithm), representative and correct biological sequences ASVs (Amplicon Sequence Variants) and the abundance table of ASVs were obtained. In QIIME2, the DADA2 plugin was used to perform quality control steps, including removing adapters, primers, and low-quality sequences (Callahan et al., 2016). The most significant alteration of QIIME2 as compared to QIIME is that DADA2 no longer conducts clustering according to sequence similarity. Instead, it merely performs dereplication, which is tantamount to clustering with 100% similarity (Bolyen et al., 2019). Then, through database species annotation and comparison, a characteristic statistical table of species classification annotation information including kingdom, phylum, class, order, family, genus, and species were acquired. An ASV accumulation curve is plotted to illustrate the impact of the number of sampled individuals on species diversity. Additionally, the rarefaction curve demonstrates that when the number of sequences is <20,000, the ASV count increases significantly with sequencing depth. Subsequently, all curves plateau, indicating sufficient data volume in this study (Figures 2, 3). Alpha diversity analysis was performed using Chao1, ACE, Shannon, and Simpson indices, with between-group comparisons conducted via Wilcoxon rank-sum test. Beta diversity analysis employed Principal Coordinates Analysis (PCoA), which reduces dimensionality of sample distance matrices (including euclidean, bray-curtis, unweighted unifrac, weighted unifrac, and jaccard distances) to identify principal components influencing microbial community composition differences. This analysis was primarily based on unweighted unifrac distance (considering species presence/absence). LEfSe analysis was conducted through three sequential steps: (1) Initial detection of significantly different species using Kruskal-Wallis rank-sum test. (2) Verification of whether all subspecies of the identified species consistently aligned to the same taxonomic level via Wilcoxon rank-sum test. (3) Final identification of biomarkers through Linear Discriminant Analysis (LDA) with thresholds set at LDA score >3 and *p* < 0.05.

## 3 Statistical analysis

SPSS 25.0 software was used to process the data. A *p* value <0.05 was considered to indicate statistically significant differences. For measurement data that conformed to a normal distribution, the Independent-Samples T test was adopted and expressed as X̄ ± S. For data that did not conform to a normal distribution, the Wilcoxon Mann–Whitney test was used and expressed as M (P_25_, P_75_). When comparing multiple sets of normally distributed data with homogeneous variances, ANOVA was adopted; for non-normally distributed data among multiple groups, the Kruskal-Wallis H test was used. For paired data (such as data before and after an intervention), if it conformed to a normal distribution, a Paired-Samples T test was used; if not, the Wilcoxon Signed-Rank test was conducted.

## 4 Results

### 4.1 General condition

The experimental group includes 48 patients, 23 males and 25 females, with a median age of 43.5 (34.0, 55.0) years and a median course of disease of 30.0 (30.0, 60.0) days. In the control group, there were 48 patients, including 26 males and 22 females, with a median age of 41.5 (33.2, 54.0) years and a median course of disease of 30.0 (15.2, 60.0) days. There were no significant differences between the experimental group and the control group in terms of gender, age, and duration of illness (*p* > 0.05). This indicates that the two groups were well-matched in these baseline characteristics, suggesting that the groups were comparable at the start of the study.

### 4.2 Efficacy of YYQFOL in treating laryngeal cough

Both the ER and the CR group showed significant improvements after treatment in their symptom and sign assessment scale scores, as well as in their LCQ scores, compared to their pre-treatment levels (*p* < 0.05). This suggests that the treatment had a positive effect on the patients’ symptoms and overall health. There was a statistically significant difference in post-treatment scores between the ER group and the CR group (*p* < 0.05). These findings indicate that the ER group demonstrated superior efficacy compared to the control intervention, and YYQFOL is effective in treating laryngeal cough with lung yin deficiency. The detailed statistical data supporting these findings can be found in Table 1.

**Table 1:** Symptom and sign scoring and LCQ.

Assessment scale	Group	Before treat	After treat	*Z*	*P* value
Symptom and sign scoring table	Experimental group	18.00 (16.00, 24.00)	7.00 (4.00, 10.00)	−5.887	<0.001
	Control group	20.00 (14.00, 26.00)	9.00 (6.00, 12.00)	−5.943	<0.001
*Z*			−2.074		
*P* value			0.038		
LCQ	Experimental group	14.53 (12.07, 16.20)	18.50 (16.98, 20.00)	−5.969	<0.001
	Control group	13.64 (11.21, 15.74)	17.48 (15.74, 18.62)	−5.867	<0.001
*Z*			−2.323		
*P* value			0.020		

### 4.3 Analysis of upper respiratory tract microbiota

#### 4.3.1 Differences between the LC and HC groups

This study involved 261 samples collected from 100 patients suffering from laryngeal cough before and after treatment and 65 healthy controls; a total of 14,440,223 sequences were mapped to 8,615 ASVs.

**Figure 4:**
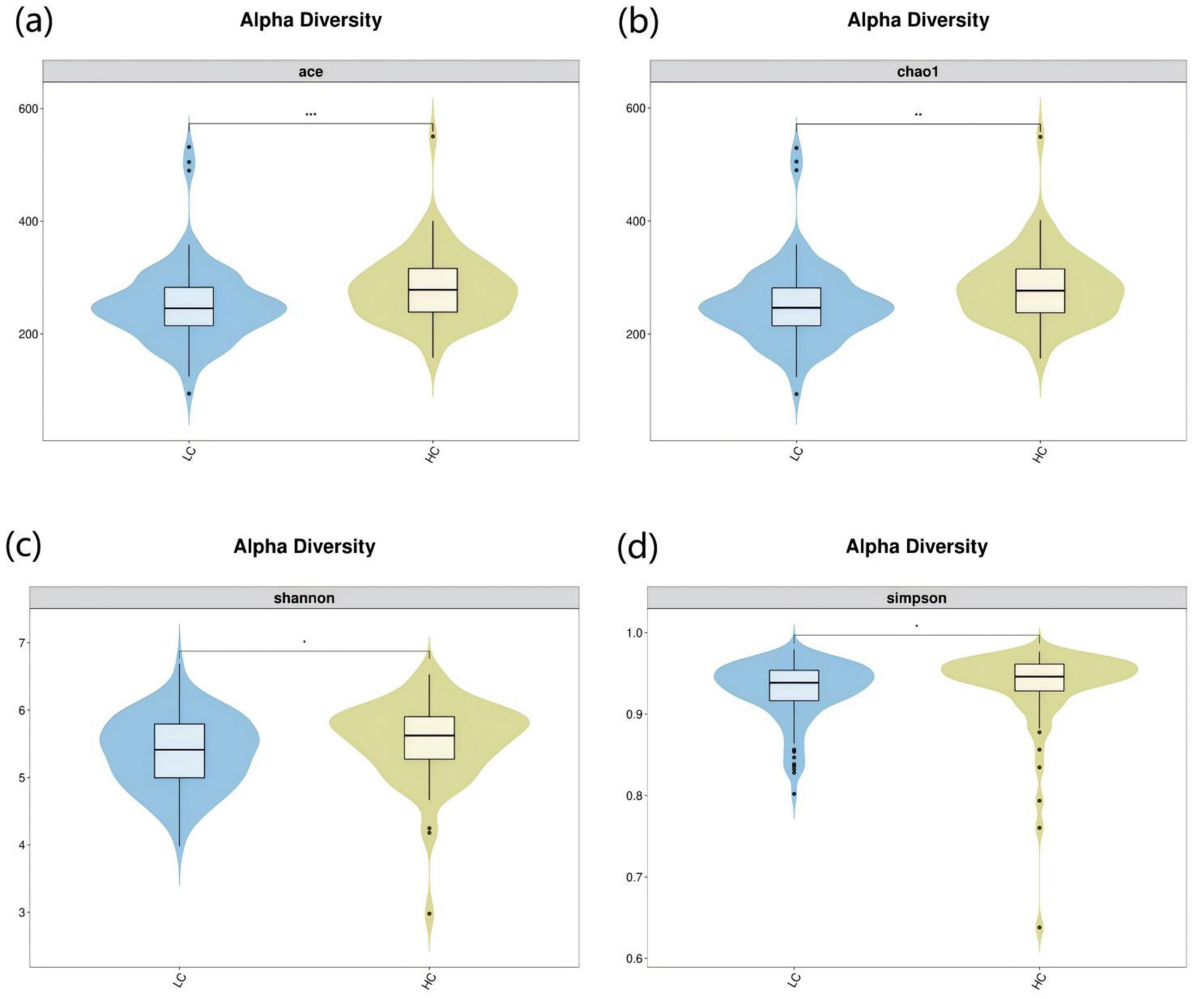
Alpha diversity analysis of LC vs HC groups. The x-axis represents different groups, and the y-axis represents the values of various indices. The vertical range reflects the dispersion degree of the index within the group, while the horizontal range indicates the data volume corresponding to the index values. • indicates denotes outliers. *indicates *P* < 0.05. **indicates *P* < 0.01. Alpha diversity analysis of the upper respiratory tract microbiota in laryngeal cough patients with LC group and HC group. The violin - box plots show data distributions. **(a)** Indicates ace index. **(b)** Indicates chao1 index. **(c)** Indicates Shannon index. **(d)** Indicates Simpson index. *P* < 0.05 was considered statistically significant.

**Figure 5:**
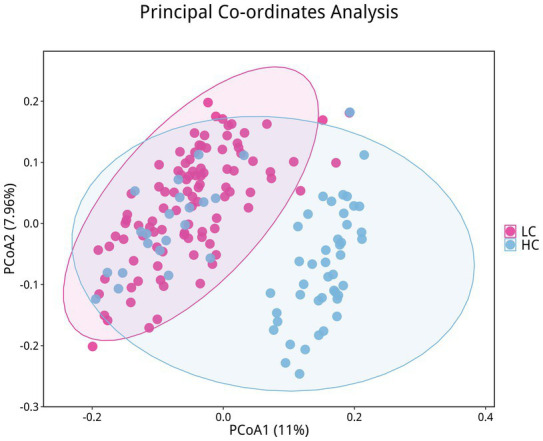
Differences in beta diversity between LC and HC groups. Points of different colors represent samples from different groups. Samples within the same group are displayed as circles (95% confidence intervals) when the number of biological replicates ≥4. Closer distances between samples indicate greater similarity in microbial community structure and smaller beta diversity.

To investigate the microbiota characteristics in laryngeal cough patients attributed to lung yin deficiency, Wilcoxon rank-sum test analysis comparing the LC and the HC group showed that all common alpha indices, such as the Ace index, Chao index, Shannon index, and Simpson index, in the upper respiratory tract microbiota were significantly lower in the LC group compared to the HC group (*p* < 0.05, Figure 4; Table 2). Furthermore, Principal Coordinate Analysis (PCoA) indicated a distinct clustering between laryngeal cough patients with lung yin deficiency and healthy controls (Figure 5).

**Table 2:** Alpha diversity of LC, HC, CR, and ER groups.

Time	Group	Ace index	Chao1 index	Shannon index	Simpson index
Before treat	HC	275.24 (238.24, 315.78)	274.50 (237.78, 315.44)	5.62 (5.24, 5.90)	0.95 (0.93, 0.96)
	LC	246.71 (215.02, 283.83)^*^	246.20 (214.69, 285.70)^*^	5.41 (4.99, 5.81)^*^	0.94 (0.92, 0.95)^*^
After treat	ER	241.95 (183.87, 283.27)^*^	241.69 (183.42, 283.33)^*^	5.3 (5.0, 5.98)	0.94 (0.91, 0.96)
	CR	218.35 (187.31, 252.86)^*^	217.79 (187.25, 252.45)^*^	5.47 (5.03, 5.66)	0.94 (0.92, 0.95)

*P < 0.05 vs. HC group.

**Figure 6:**
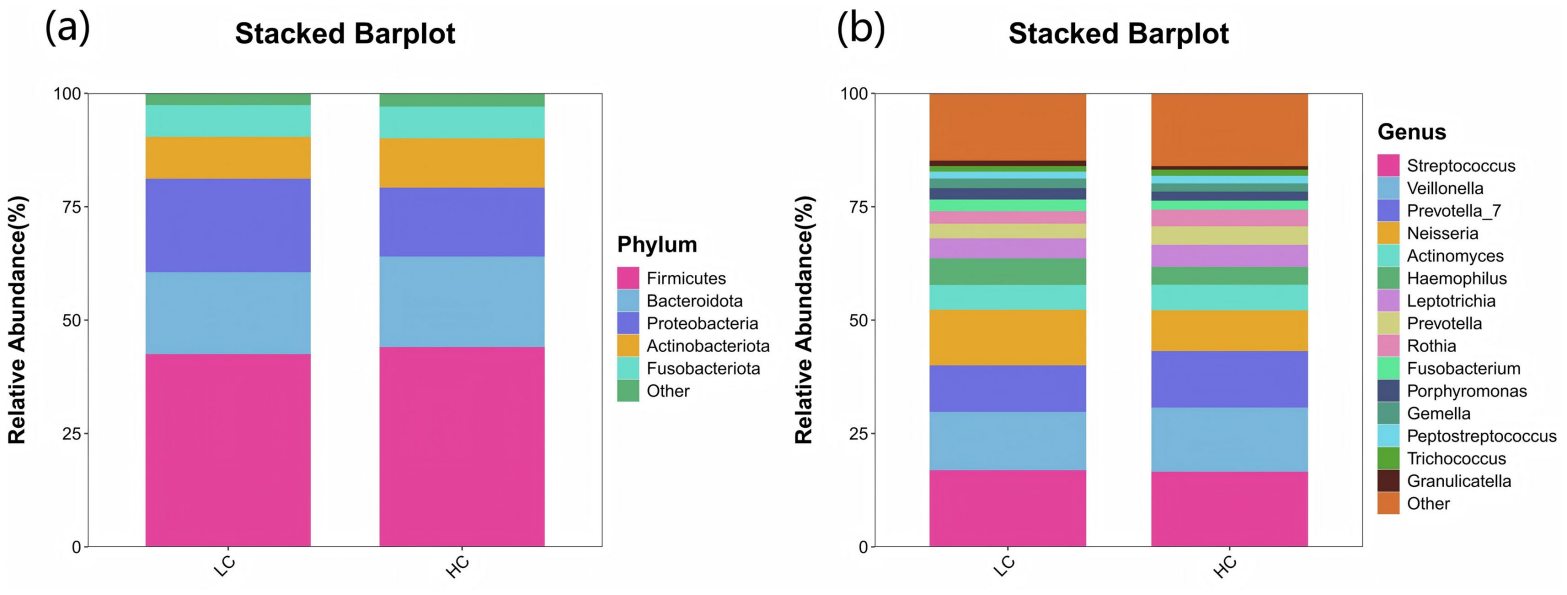
Species distribution between LC and HC groups. The x-axis represents the groups, while the y-axis indicates the relative abundance of taxonomic classifications. Different colors correspond to distinct species at the same taxonomic level. The bar plot clearly displays the dominant species composition within each group and facilitates comparison of abundance trends across different groups. **(a)** The species composition of the LC and HC groups at the phylum level is dominated by the top 5 phyla, including Firmicutes, Bacteroidota, Proteobacteria, Actinobacteriota, and Fusobacteriota. **(b)** At the genus level, the composition is represented by the top 15 genera, such as Streptococcus, Veillonella, Prevotella_7, Neisseria, and Actinomyces.

In terms of microbial composition, *Firmicutes*, *Bacteroidota*, *Proteobacteri*, *Actinobacteriota*, and *Fusobacteriota* were the predominant phyla in all samples. The proportion of some phyla in the LC group before treatment was significantly lower than that in the HC group, including *Actinobacteriota*, *Patescibacteria*, *Cyanobacteria*, and *Verrucomicrobiota* (*p* < 0.05). In contrast, the proportion of *Proteobacteria* before treatment was significantly higher (*p* < 0.05), as shown in Figure 6a. At the genus level, the predominant genera in both LC and HC groups included *Streptococcus*, *Veillonella*, *Prevotella_7*, *Neisseria*, *Actinomyces*, and *Haemophilus*. And the research revealed that *Neisseria*, *Haemophilus*, and *Fusobacterium* were significantly enriched in LC patients compared to healthy controls (*p* < 0.05), as shown in Figure 6b.

**Figure 7:**
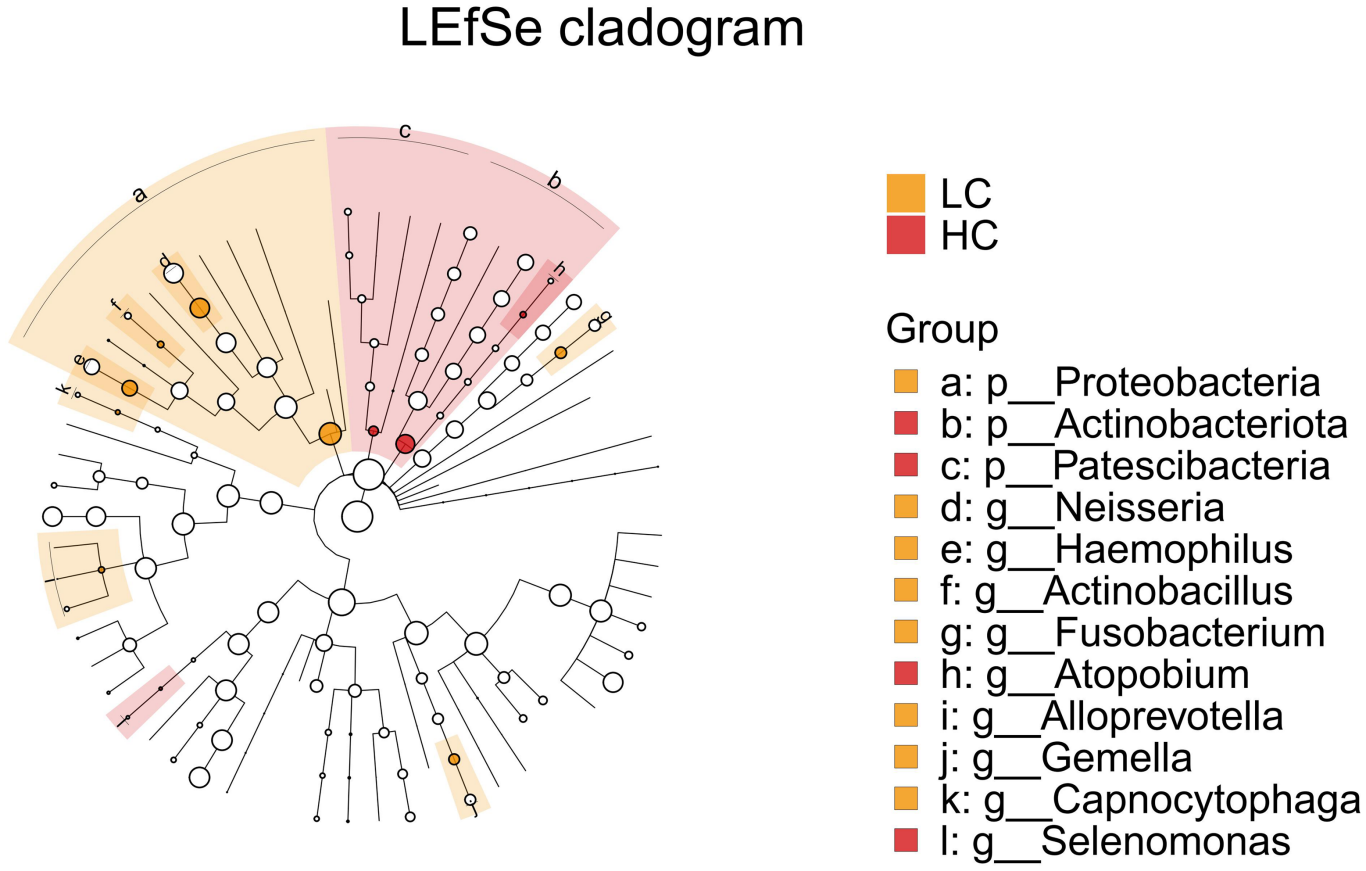
LEfSe and barplot cladogram of LC vs HC groups. In the cladogram, concentric circles from center to periphery represent taxonomic levels from phylum to species. Circle sizes at each level are proportional to relative abundance. Taxa without significant differences remain uncolored, while differentially abundant taxa are colored according to their dominant group (color-coded by experimental groups). Nodes highlight key microbial biomarkers within respective groups.

LEfSe (LDA Effect Size) analysis was employed to compare two or more groups, aiming to identify species (biomarkers) with significant differences in abundance among the groups. Proteobacteria, Actinobacteriota, Neisseria, Haemophilus, Actinobacillus, Fusobacterium display a statistically significant difference in abundance between the LC and HC groups, as shown in Figure 7. Notably, Actinobacillus was found to be enriched in the upper respiratory tract samples of healthy controls, indicating it may play a significant role in maintaining the microbial balance in respiratory system of healthy subjects.

#### 4.3.2 The efficacy of Yangyin Qingfei oral liquid

**Figure 8:**
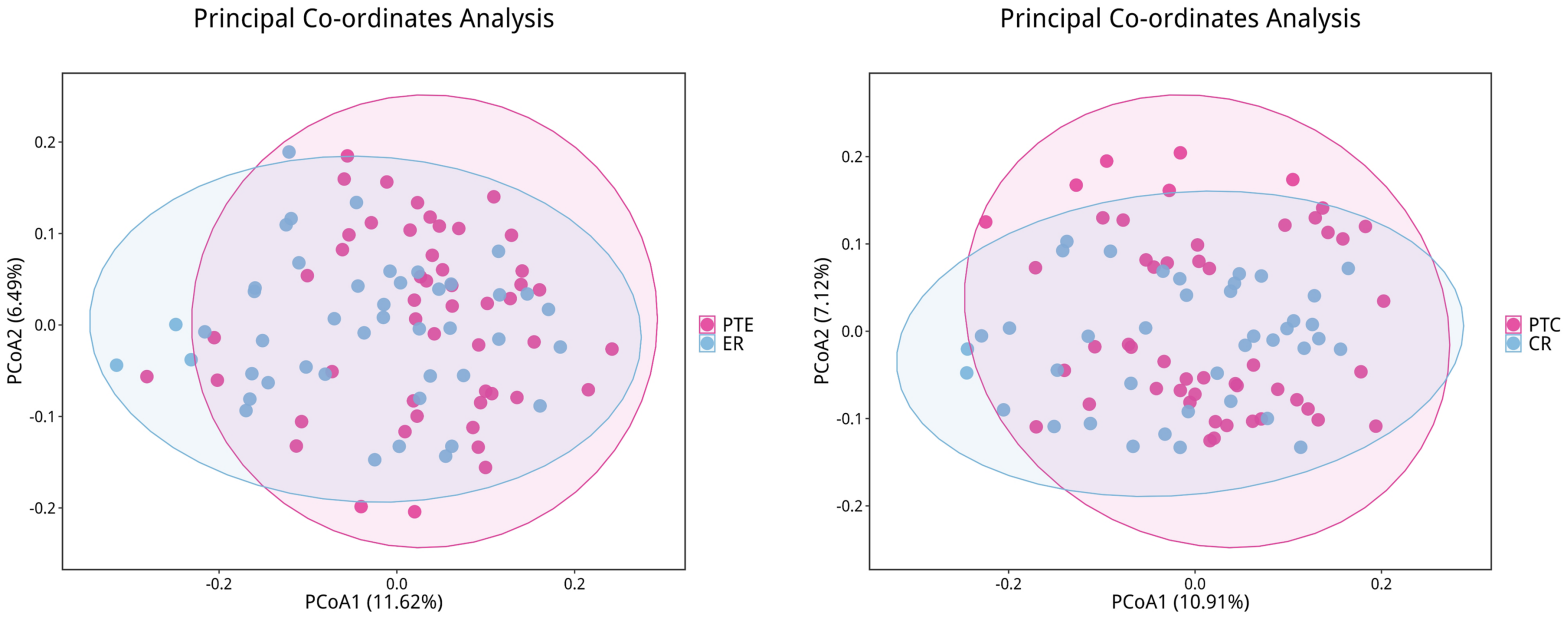
Differences in beta diversity between PTE/PTC and ER/CR groups. Points of different colors represent samples from different groups. Samples within the same group are displayed as circles (95% confidence intervals) when the number of biological replicates ≥4. Closer distances between samples indicate greater similarity in microbial community structure and smaller beta diversity.

**Figure 9:**
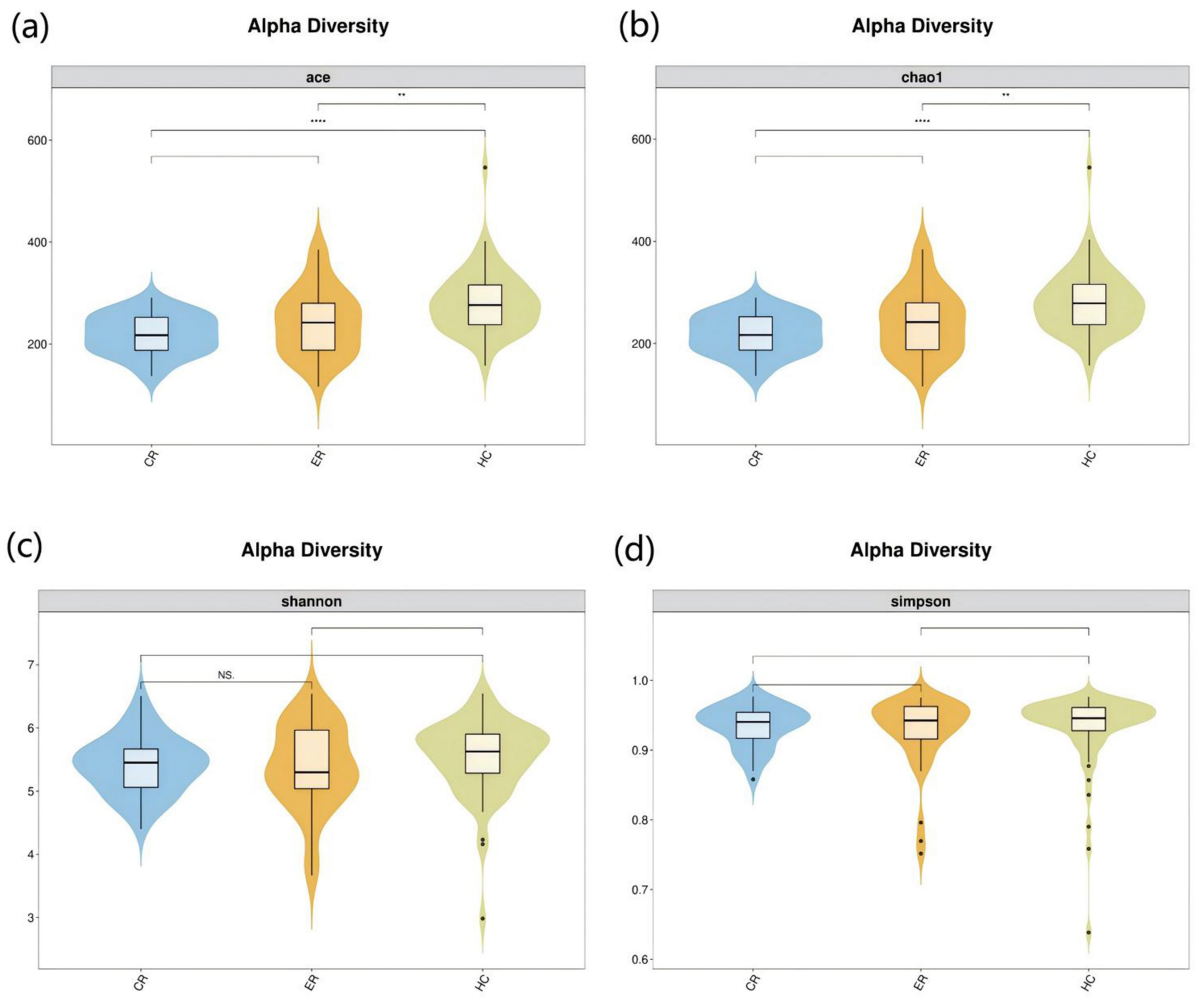
Alpha diversity analysis among the CR, ER, and HC groups. The x-axis represents different groups, and the y-axis represents the values of various indices. The vertical range reflects the dispersion degree of the index within the group, while the horizontal range indicates the data volume corresponding to the index values. •indicates denotes outliers. NS indicates *P* > 0.05 (not significant). **indicates *P* < 0.01. **** indicates *P* < 0.001. Alpha diversity analysis of the upper respiratory tract microbiota in laryngeal cough patients with CR, ER and HC groups. The violin - box plots show data distributions. **(a)** Indicates ace index. **(b)** Indicates chao1 index. **(c)** Indicates Shannon index. **(d)** Indicates Simpson index. *P*< 0.05 was considered statistically significant.

**Figure 10:**
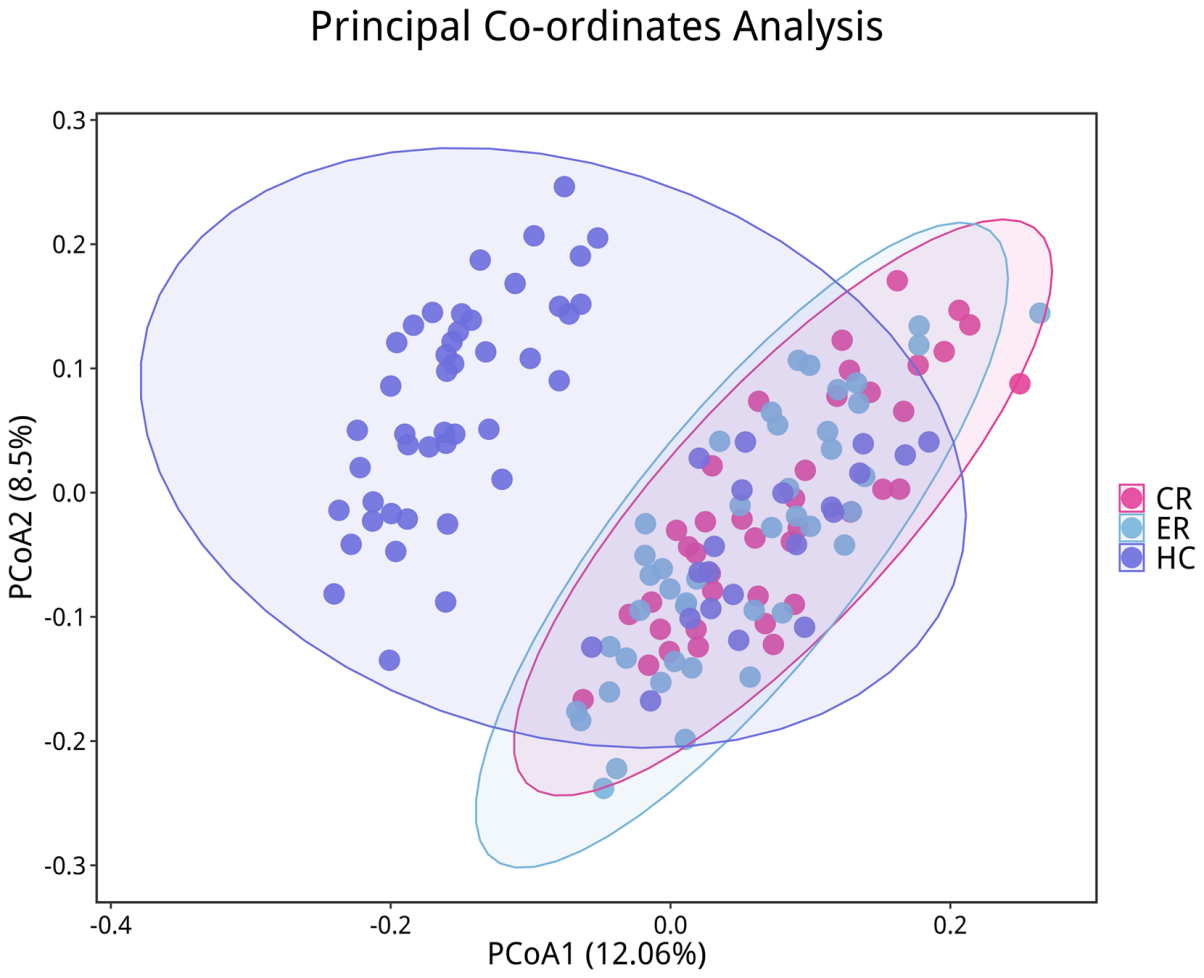
Differences in beta diversity among the CR, ER, and HC groups. Points of different colors represent samples from different groups. Samples within the same group are displayed as circles (95% confidence intervals) when the number of biological replicates ≥4. Closer distances between samples indicate greater similarity in microbial community structure and smaller beta diversity.

To analyze the efficacy of YYQFOL, we compared the PTE with the ER group, as well as the PTC with the CR group. No significant differences were observed in alpha diversity analysis (*p* > 0.05, Table 3), whereas beta diversity analysis showed statistically significant differences (*p* < 0.05), as shown in Figure 8. From the perspective of community richness, statistically significant differences were observed in the Chao1 and ACE indices among the ER, CR, and HC groups. Specifically, significant differences were found between the ER group and the HC group, as well as between the CR group and the HC group (*p* < 0.05). However, no statistically significant difference was observed between the ER and CR groups (*p* > 0.05). In terms of community diversity, no statistically significant differences were found in the Shannon or Simpson indices among the three groups (*p* > 0.05, Figure 9; Table 2). In PCoA, the Figure 10 showed significant differences in species composition among the CR, ER, and HC groups.

**Table 3:** Alpha diversity before and after treatment.

Group	Chao1 index	Ace index	Shannon index	Simpson index
PTC	196.85 (226.33, 256.27)	196.32 (225.85, 257.14)	4.88 (5.35, 5.80)	0.91 (0.94, 0.95)
CR	187.25 (217.79, 252.45)	187.31 (218.35, 252.86)	5.03 (5.47, 5.66)	0.92 (0.94, 0.95)
*P*	0.166	0.143	0.687	0.619
PTE	233.22 (259.80, 294.91)	234.01 (259.34, 294.29)	4.99 (5.47, 5.70)	0.91 (0.94, 0.95)
ER	183.42 (241.69, 283.33)	183.87 (241.95, 283.27)	5.00 (5.30, 5.98)	0.91 (0.94, 0.96)
*P*	0.105	0.102	0.981	0.608

**Figure 11:**
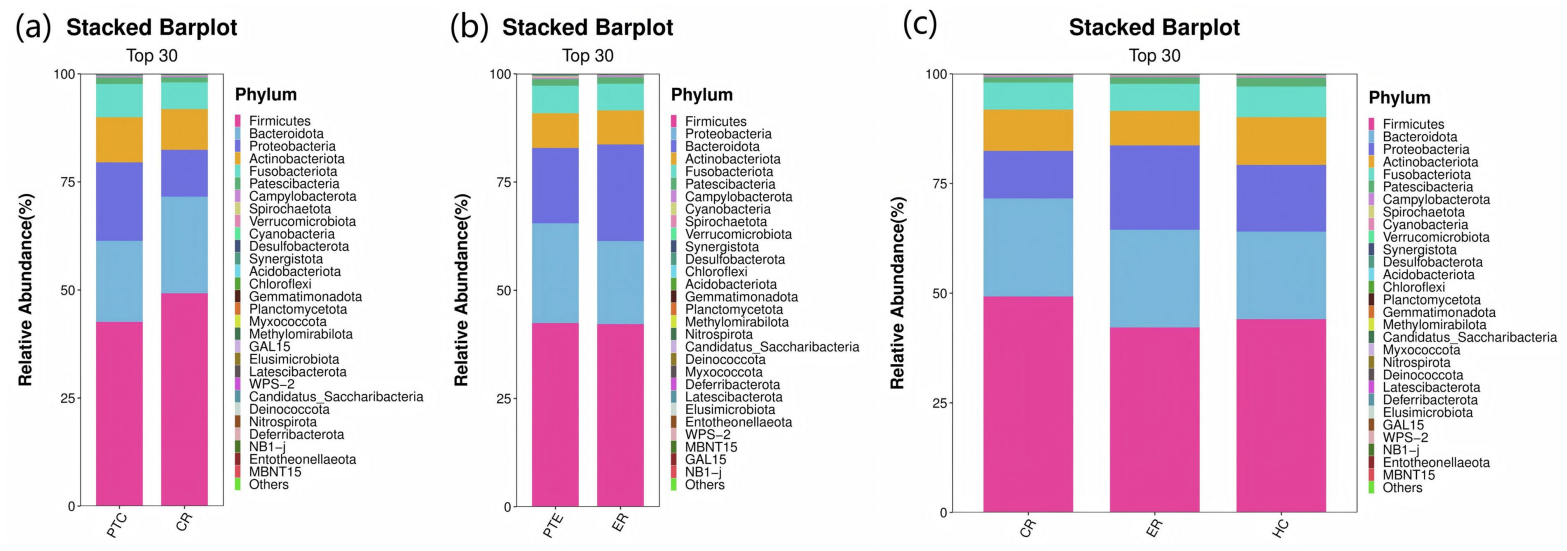
**(a)** Phylum-level species distribution of PTC vs CR groups. **(b)** Phylum-level species distribution of PTE vs ER groups. **(c)** Phylum-level species distribution among the CR, ER, and HC groups.

**Figure 12:**
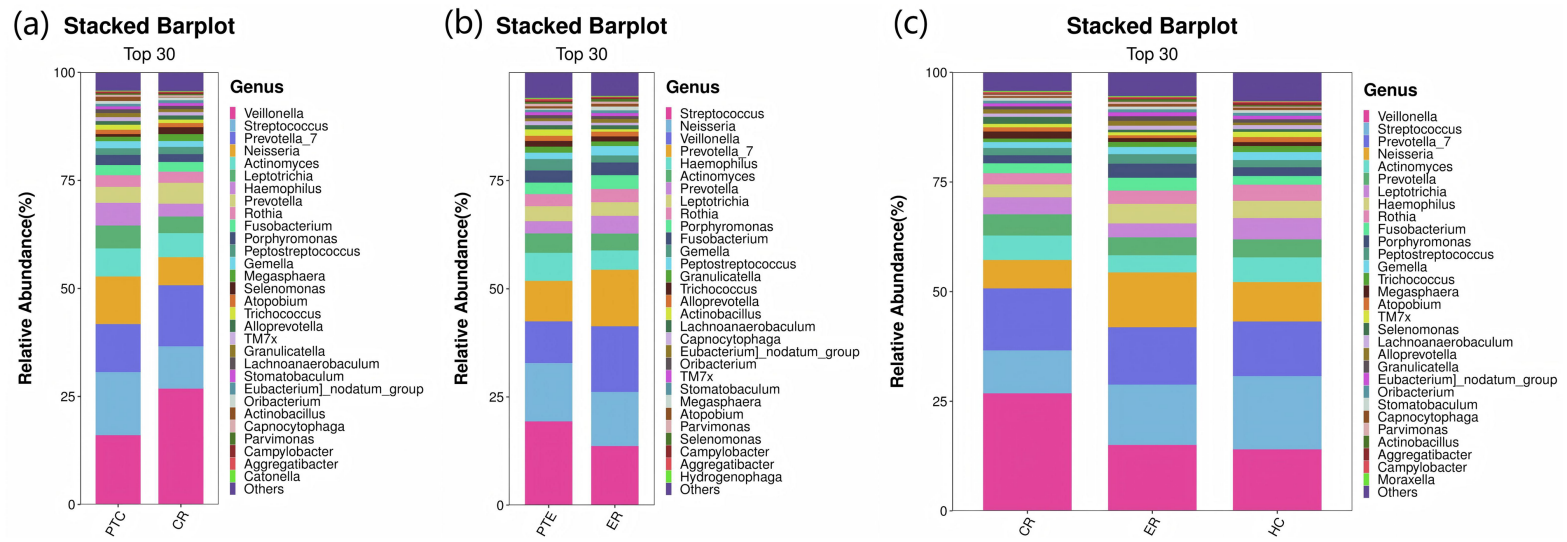
**(a)** Genus-level species distribution of PTC vs CR groups. **(b)** Genus-level species distribution of PTE vs ER groups. **(c)** Genus-level species distribution among the CR, ER, and HC groups.

At the phylum level, compared to pre-treatment levels, the control group exhibited significant increases in *Firmicutes* and *Bacteroidota* (*p* < 0.05), as well as significant decreases in *Proteobacteria* and *Verrucomicrobiota* post-treatment (*p* < 0.05), as shown in Figure 11a. In the experimental group, *Proteobacteria* exhibited a significant decline post-treatment compared to pre-treatment levels (*p* < 0.05), as shown in Figure 11b. Statistically significant differences were observed among the CR, ER, and HC groups in the abundance of *Firmicutes*, *Proteobacteria*, *Actinobacteriota*, *Patescibacteria*, *Cyanobacteria*, and *Verrucomicrobiota* (*p* < 0.05). Specifically, comparisons revealed significant differences between the ER and CR groups in *Firmicutes* and *Proteobacteria* abundances (*p* < 0.05). The ER group had significantly lower *Firmicutes* and higher *Proteobacteria* levels than the CR group (*p* < 0.05). Additionally, between the CR and HC groups in *Firmicutes*, *Patescibacteria*, and *Verrucomicrobia*, as well as between the ER and HC groups in *Actinobacteria*, *Cyanobacteria*, and *Verrucomicrobia*, statistically significant differences were observed (*p* < 0.05), as shown in Figure 11c. At the genus level, compared to pre-treatment, the control group showed significant post-treatment increases in *Veillonella* and *Prevotella_7* (*p* < 0.05), alongside decreases in *Streptococcus*, *Neisseria*, and *Haemophilus* (*p* < 0.05), as shown in Figure 12a. Similarly, the experimental group exhibited elevated *Veillonella* and *Prevotella_7* levels (*p* < 0.05) and reduced *Streptococcus* and *Haemophilus* levels post-treatment (*p* < 0.05), as shown in Figure 12b. Statistically significant differences were observed in *Veillonella*, *Streptococcus*, *Actinomyces*, and *Leptotrichia* among the CR, ER, and HC groups (*p* < 0.05). Specifically, the ER and CR groups differed significantly in *Veillonella* abundance, with the ER group displaying markedly lower levels than the CR group (*p* < 0.05). Additionally, the CR and HC groups differed significantly in *Veillonella* and *Streptococcus* (*p* < 0.05). The ER and HC groups exhibited significant differences in *Leptotrichia* (*p* < 0.05), as shown in Figure 12c. It can be seen from this that, compared with the control group, the distribution of flora in the experimental group after medication was closer to that of healthy people.

**Figure 13:**
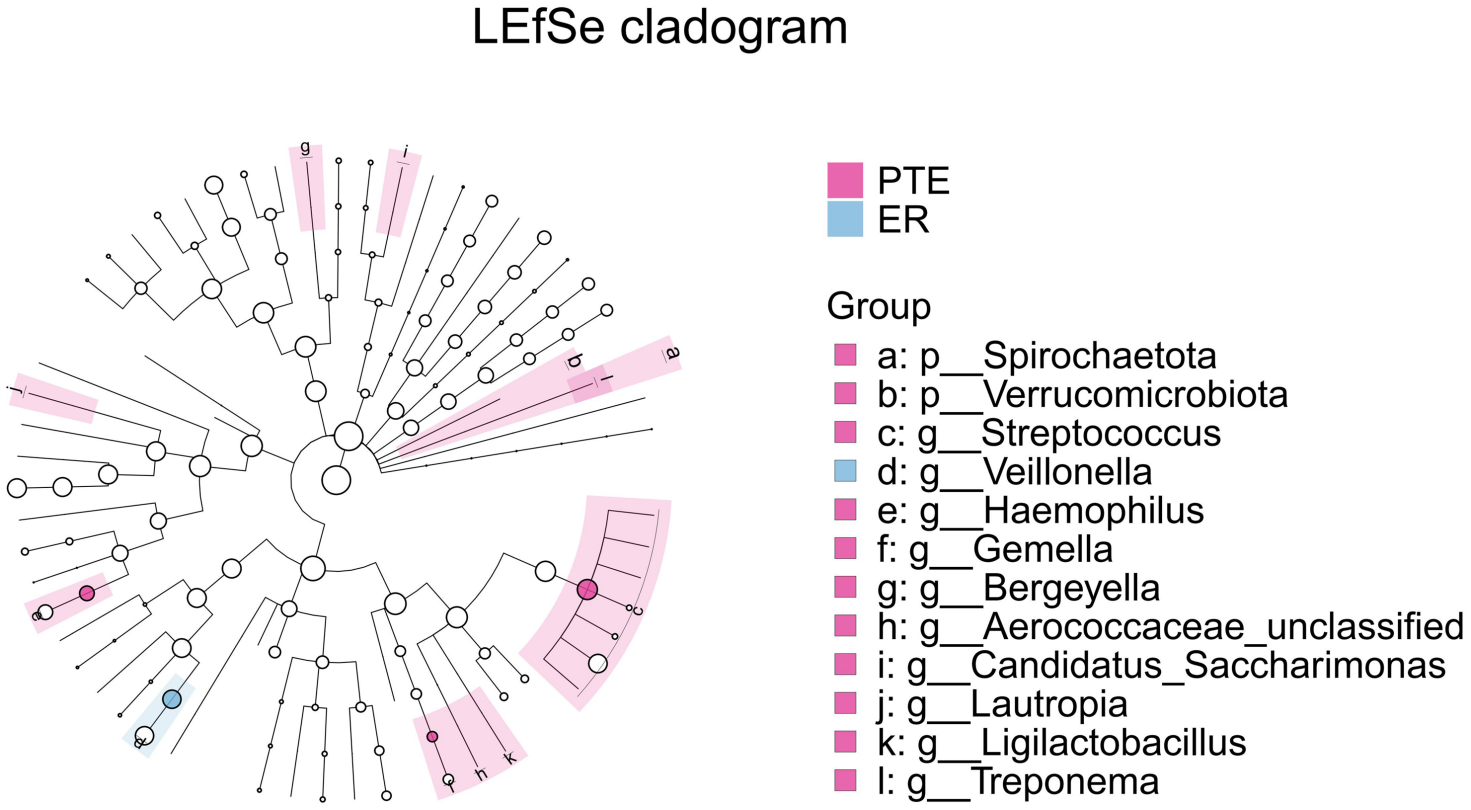
LEfSe and barplot cladogram of PTE vs ER groups. In the cladogram, concentric circles from center to periphery represent taxonomic levels from phylum to species. Circle sizes at each level are proportional to relative abundance. Taxa without significant differences remain uncolored, while differentially abundant taxa are colored according to their dominant group (color-coded by experimental groups). Nodes highlight key microbial biomarkers within respective groups.

**Figure 14:**
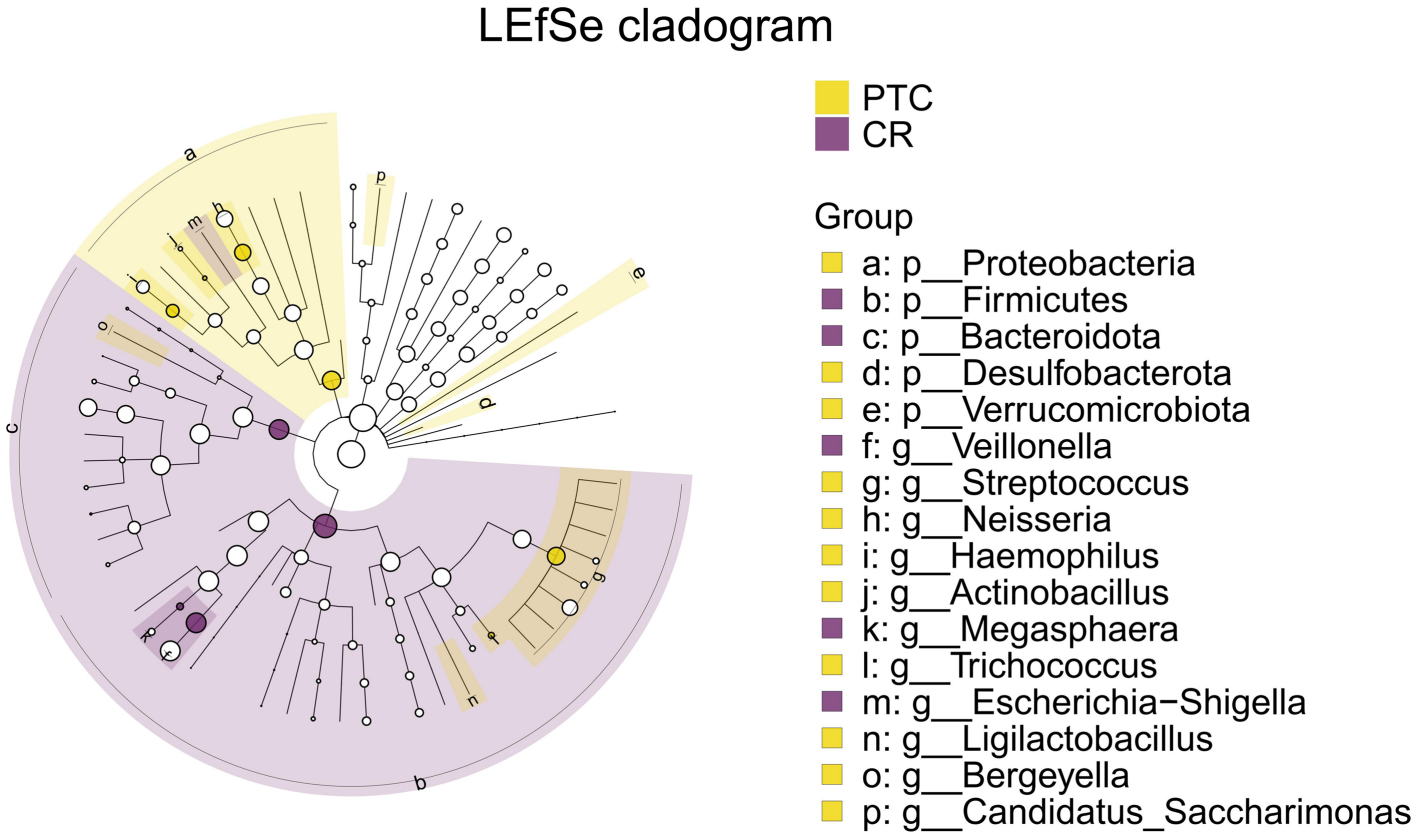
LEfSe and barplot cladogram of PTC vs CR groups. In the cladogram, concentric circles from center to periphery represent taxonomic levels from phylum to species. Circle sizes at each level are proportional to relative abundance. Taxa without significant differences remain uncolored, while differentially abundant taxa are colored according to their dominant group (color-coded by experimental groups). Nodes highlight key microbial biomarkers within respective groups.

The intersection of differential analysis results at the genus level revealed that the following genera exhibited significant differences between the PTE and ER groups: *Streptococcus*, *Veillonella*, *Haemophilus*, and *Gemella*, as shown in Figure 13. Specially, *Veillonella* was enriched in the ER group, while the remaining genera were enriched in the PTE group. The overlap of differential analyses at phylum and genus levels showed significant differences between PTC and CR groups, including *Proteobacteria*, *Firmicutes*, *Streptococcus*, *Veillonella*, and *Neisseria*, as shown in Figure 14. Specially, *Firmicutes* and *Veillonella* were enriched in the CR group, while *Proteobacteria*, *Streptococcus*, and *Neisseria* were enriched in the PTC group. The intersection of the differential microbiota before and after treatment in the experimental group and the control group showed significant differences in *Streptococcus* and *Veillonella*. The experimental group uniquely showed differences in *Haemophilus*.

**Figure 15:**
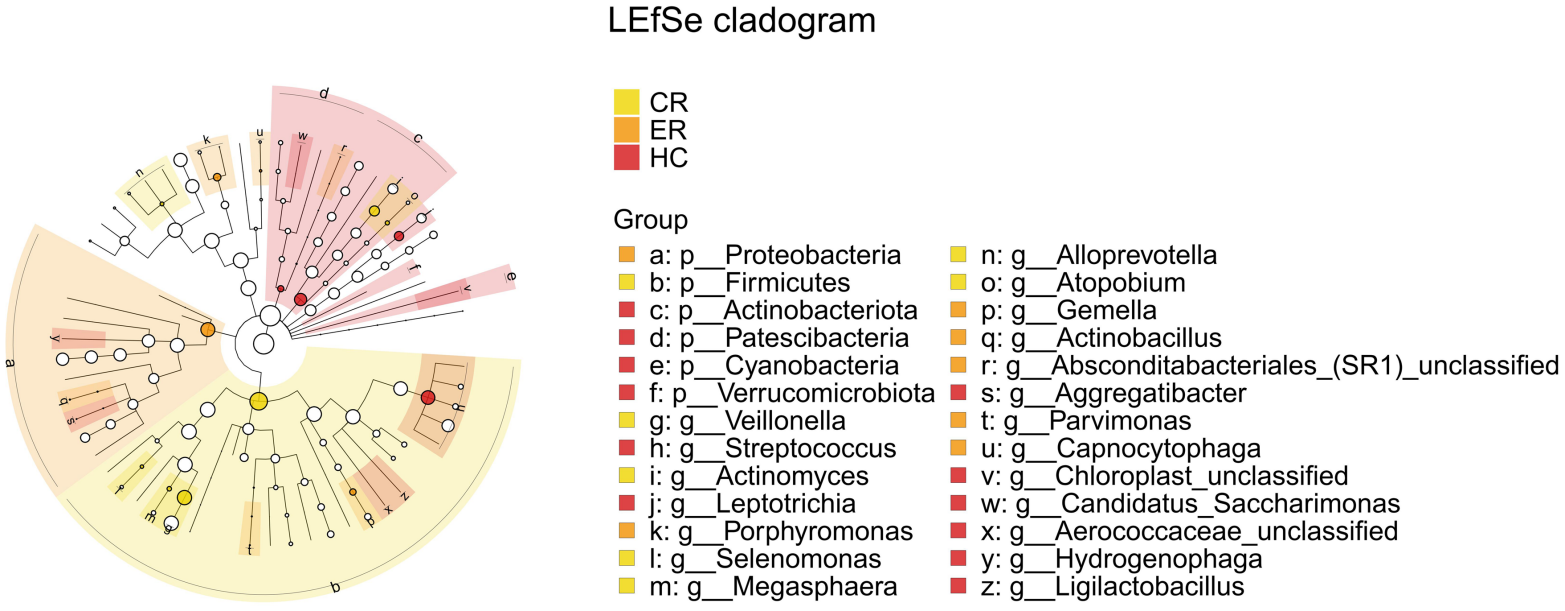
LEfSe and barplot cladogram the among the CR, ER, and HC groups. In the cladogram, concentric circles from center to periphery represent taxonomic levels from phylum to species. Circle sizes at each level are proportional to relative abundance. Taxa without significant differences remain uncolored, while differentially abundant taxa are colored according to their dominant group (color-coded by experimental groups). Nodes highlight key microbial biomarkers within respective groups.

Comparative analysis of the ER, CR, and HC groups identified significant differences in the relative abundance of *Firmicutes*, *Proteobacteria*, *Actinobacteria*, *Veillonella*, and *Streptococcus*, as shown in Figure 15. Specifically, *Firmicutes* and *Veillonella* abundances were significantly decreased in the ER group compared to the CR group, while *Proteobacteria* abundance showed a significant increase. Comparative analysis revealed significant differences in *Firmicutes*, *Veillonella*, and *Streptococcus* between the CR and HC groups, and in *Actinobacteria* between the ER and HC groups.

#### 4.3.3 Differences between the responders and non-responders

**Figure 16:**
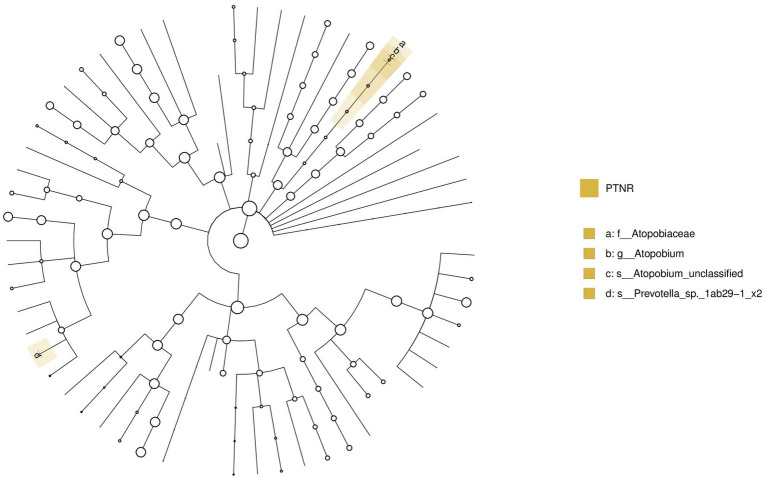
LEfSe and barplot cladogram of PTR vs PTNR groups. In the cladogram, concentric circles from center to periphery represent taxonomic levels from phylum to species. Circle sizes at each level are proportional to relative abundance. Taxa without significant differences remain uncolored, while differentially abundant taxa are colored according to their dominant group (color-coded by experimental groups). Nodes highlight key microbial biomarkers within respective groups.

A comparative analysis was conducted between the pre-treatment responders (PTR) and pre-treatment non-responders (PTNR). Analysis of microbiota differences between the PTR and PTNR groups identified *Atopobium* as a distinct bacterial genus, as shown in Figure 16. This genus was significantly enriched in the PTNR group, suggesting that its abundance might correlate with the degree of clinical improvement in patients.

## 5 Discussion

Through the analysis, we discovered an imbalance in upper respiratory tract flora in patients with laryngeal cough when compared to healthy controls. Indices such as the Ace index, Chao index, Shannon index, and Simpson index, which measure the abundance and diversity of the respiratory microbiota, were found to be lower in laryngeal cough patients than in healthy subjects. This indicates that the diversity and abundance of respiratory microbiota in laryngeal cough patients are reduced compared to healthy controls. PCoA analysis indicated that the species composition and abundance of the microbial community in the LC group showed apparent similarity to those in the HC group, though discrepancies existed in low-abundance species between the two groups. This suggests that laryngeal cough primarily influences the upper respiratory tract microbiota by modulating the abundance of dominant species and triggering adaptive adjustments in the dominant microbial community, while concurrently facilitating the proliferation of opportunistic infectious bacteria. Fund Human Microbiome Project revealed that the throat microbiome exhibits distinct taxonomic and OTU-level features. At the genus level, it is dominated by common oral genera such as *Streptococcus*, *Fusobacterium*, and *Veillonella*, which are part of the core microbiome shared across multiple oral sites (Huse et al., 2012). Human larynx is dominated by *Proteobacteria*, *Firmicutes*, *Bacteroidetes*, *Actinobacteria*, and *Fusobacteria* (Hanshew et al., 2014), which is consistent with our research on the distribution characteristics of upper respiratory tract flora in patients with laryngeal cough. The larynx, a mucosal organ at the junction of the respiratory and digestive tracts, serves as a shared pathway for air and food, creating a unique niche with selective microbial colonization pressure. A gnotobiotic mouse study shows gut microbiota can selectively colonize the larynx, forming communities similar to those in conventionally raised mice, with dominant genera like *Streptococcus* and *Lactobacillus* reflecting the organ’s ecological specificity and functional adaptation to its dual-system role (An et al., 2020).

Our study showed that genera *Streptococcus*, *Veillonella*, and *Haemophilus* exhibited differences following treatment with YYQFOL. PICRUSt2 analysis indicated significant differences in metabolic functions among the ER, CR, and HC microbiomes. However, the functional data derived from PICRUSt2 cannot serve as a substitute for metagenomic, metatranscriptomic, or metabolomic investigations. Experimental validation is required to prove our predictions. A study constructed a 3D vocal fold mucosa model, highlighted the opposing roles of pathogenic and commensal bacteria in vocal fold mucosal health, with *Streptococcus pseudopneumoniae* driving pathological remodeling and *Streptococcus salivarius* contributing to epithelial maintenance, offering insights into microbial mechanisms in laryngeal diseases (Lungova et al., 2024). Research has indicated that laryngeal cough is closely associated with dysbiosis of the respiratory microbiota (Dąbrowska et al., 2020). Patients suffering from laryngeal cough often show a significant reduction in microbial diversity in the larynx. This is characterized by an increased presence of pathogenic bacteria such as *Haemophilus*, while beneficial commensal bacteria like *Streptococcus*, *Prevotella*, and *Veillonella* are markedly reduced. This imbalance disrupts mucosal homeostasis and fosters chronic inflammation, contributing to cough hypersensitivity (Budden et al., 2019).

We selected patients with laryngeal coughs attributed to lung yin deficiency as research subjects, and the YYQFOL demonstrated good therapeutic efficacy. Our results showed that treatment with YYQFOL significantly decreased the proportions of Bergeyella, Granulicatella, and Lautropia in the upper respiratory tract microbiota. Relative to healthy controls, the CR group exhibited a significant reduction in Streptococcus (*p* < 0.05) and an increase in Veillonella (*p* < 0.05), while the experimental group’s microbiota closely mirrored those of healthy individuals.

Studies have demonstrated that the components of YYQFOL exhibit multiple pharmacological actions. Ingredients such as Licorice, Bulbus Fritillariae Cirrhosae and Ophiopogonis tuber possess anti-inflammatory effects, which help alleviate airway inflammation and reduce cough triggered by inflammatory stimuli (Yang et al., 2017; Wang et al., 2011; Ishibashi et al., 2001). Meanwhile, Menthone modulate the Th1/Th2 immune balance, thereby diminishing airway hyperresponsiveness (Su and Lin, 2022). By regulating immune function and enhancing the body’s disease resistance, this formulation effectively reduces the frequency of cough episodes.

Our analysis and comparison of the upper respiratory tract microbiota in patients with laryngeal cough and healthy controls revealed that laryngeal cough patients exhibited dysbiosis, characterized by lower richness and diversity in their microbiota compared to healthy controls. These findings suggest that the pathogenesis of laryngeal cough is closely associated with an imbalance in the upper respiratory tract microbiota. Treatment with YYQFOL can increase both the diversity and abundance of this microbiota, playing a key therapeutic role in improving the structure of the respiratory microbiota and maintaining the dynamic balance of bacterial communities.

However, the specific mechanisms by which YYQFOL regulates the upper respiratory tract microecology still require further investigation. Our study provides new experimental evidence and therapeutic strategies for laryngeal cough. Future research needs to explore these mechanisms to provide new insights for the comprehensive treatment of laryngeal cough.

## Data Availability

The original contributions presented in the study are publicly available. This data can be found here: https://www.ncbi.nlm.nih.gov/, accession number: PRJNA1237712.
